# The genus *Spathius* Nees (Hymenoptera, Braconidae, Doryctinae) in Mexico: occurrence of a highly diverse Old World taxon in the Neotropics

**DOI:** 10.3897/zookeys.427.8074

**Published:** 2014-07-21

**Authors:** Sergey A. Belokobylskij, Alejandro Zaldívar-Riverón

**Affiliations:** 1 Zoological Institute, Russian Academy of Sciences, St Petersburg 199034, Russia; 2 Museum and Institute of Zoology PAN, Wilcza 64, 00-679 Warsaw, Poland; 3 Colección Nacional de Insectos, Instituto de Biología, Universidad Nacional Autónoma de México, 3er. circuito exterior s/n, Cd. Universitaria, Copilco, Coyoacán, A. P. 70–233, C. P. 04510., D. F., México

**Keywords:** Ectoparasitoid, Central America, Ichneumonoidea, taxonomy, new species

## Abstract

Two new species of the parasitoid wasp genus *Spathius* Nees (Braconidae: Doryctinae) from Mexico, *S. mexicanus* **sp. n.** and *S. chamelae* **sp. n.**, are described and illustrated. These represent the second and third described species of this highly diverse Old World genus in the Neotropics, and the first described species recorded for the Mexican territory.

## Introduction

The parasitoid wasps genus *Spathius* Nees, 1818 (Braconidae: Doryctinae) is a highly diverse group that currently contains about 400 described species divided into 40 species groups (Nixon 1943, Belokobylskij 2003, Chen and Shi 2004, Belokobylskij and Maeto 2009). This genus is widely distributed along the five continents, though most of its species richness concentrates in the Oriental and Palaearctic regions. Most species of *Spathius* whose biology is known are idiobiont ectoparasitoids of the xylophagous larvae of various coleopteran families, particularly Curculionidae, Cerambycidae, Anobiidae, Bostrichidae and Buprestidae. However, some species have been also reared from concealed larvae of Lepidoptera (Sessiidae, Tineidae, Pyralidae and Tortricidae) and Hymenoptera (Xiphydriidae) (Shenefelt and Marsh 1976, Belokobylskij 1992, 2003, Yu et al. 2012).

A molecular phylogenetic study carried out for 50 doryctine genera (Zaldívar-Riverón et al. 2008) revealed that the tribe Spathiini *sensu* Belokobylskij (1992), whose members had been mainly characterised by having a tubular, considerably elongated acrosternite (basal sternal plate) of the first metasomal segment, was polyphyletic. Based on the phylogenetic relationships recovered, these authors left the Spathiini to be composed only by the speciose *Spathius*, pendant to the inclusion of additional doryctine genera in further phylogenetic studies.

The most recent revision for the Nearctic species of *Spathius* registered a total of 18 species (Marsh and Strazanac 2009). In contrast, only one species of this genus has been recorded to occur in the Neotropics, *Spathius albocoxus* Marsh, described from Costa Rica (Marsh 2002). Recent studies have reported the occurrence of species assigned to *Spathius albocoxus* in the state of Yucatan, Mexico (Cauich-Kumul et al. 2012; Coronado-Blanco 2013). However, these identifications apparently followed Marsh’s (2002) key but without any further detailed character examination and thus they are questionable.

In this paper, we described two new species of *Spathius* from two localities situated near the Pacific and Atlantic coasts of Mexico. These are the first confirmed records of the genus in the Mexican territory as well as two additional species of *Spathius* described for the Neotropics. According of Nixon’s (1943) key, these two new species belong to the *Spathius fasciatus* species group.

## Material and methods

The examined material was collected by AZR in the Chamela (state of Jalisco) and Los Tuxtlas (state of Veracruz) biological stations, both owned by the Instituto de Biología, Universidad Nacional Autónoma de México (IB-UNAM). The type material of the new species of *Spathius* described below is deposited in the following collections: IB-UNAM – Colección Nacional de Insectos, Instituto de Biología, Universidad Nacional Autónoma de México (México); ZISP – Zoological Institute, Russian Academy of Sciences (St Petersburg, Russia).

The terminology employed for morphological features and measurements follows Belokobylskij and Maetô (2009). The wing venation nomenclature follows Belokobylskij and Maetô (2009), with Sharkey and Wharton’s (1997) terminology shown in parentheses. Photographs were taken with a Leica IC 3D digital camera that was mounted on a Leica® MZ16 microscope and using the Leica Application Suite® imaging system (Museum and Institute of Zoology PAN, Warsaw, Poland).

DNA sequences belonging to the barcoding locus (about 658 bp of the cytochrome oxidase I mitocondrial DNA gene) were generated for specimens of the two new species, and their GenBank numbers are included below. Sequences of the species from Chamela, Jalisco, were previously published (Zaldívar-Riverón et al. 2010), and are also available in the project file “Parasitoid Wasps (Braconidae: Doryctinae) of Chamela–Cuixmala Biosphere Reserve” (ASDOR project) in the projects section of the Barcode of Life Data Systems (www.barcodinglife.org). Sequences of the specimens from Los Tuxtlas, Veraruz, were obtained using the DNA extraction and amplification protocols described in Ceccarelli et al. (2012).

## Taxonomic part

### Spathius mexicanus

Taxon classificationAnimaliaHymenopteraBraconidae

Belokobylskij & Zaldívar-Riverón sp. n.

http://zoobank.org/6833714C-2FA6-4B9B-936A-C8BD036D51BC

Figs 1 –16

#### Type material.

*Holotype*: female, “México, Veracruz, Los Tuxtlas, 27.VII.2006, A. Zaldívar-Riveron coll.”, DNA voucher no. CNIN1193, GenBank accession no. KM099422 (IB-UNAM).

*Paratypes*: 1 male “México, Veracruz. Estación de Biología Los Tuxtlas, 18.585N, 95.075W, 151msnm, 09.VI.2011. Red. Remanente selva alta perennifolia. Col. Zaldívar-Riverón, Clebsch, Martínez-Salinas”, DNA voucher no. CNIN1196, Genbank accession no. KM099423 (IB-UNAM); 1 female, “México, Veracruz, Estación de Biología Los Tuxtlas, 27.VII.2006” (ZISP); 1 female, 5 males, “México, Veracruz. Estación de Biología Los Tuxtlas, 18.585N, 95.074W, 141 msnm, 08.VI.2011. Tropical rain forest. Col. Zaldívar-Riverón, Clebsch, Martínez-Salinas” (IB-UNAM, ZISP); 2 females, 1 male, “México, Veracruz. Estación de Biología Los Tuxtlas, 18.585N, 95.075W, 151 msnm, 09.VI.2011. Col. Zaldívar-Riverón, Clebsch, Martínez-Salinas, Selva altas” (IB-UNAM); 4 females, “México, Veracruz. Estación de Biología Los Tuxtlas, 18.58512N, 95.075W, 7–11 m, 12.VI.2011. Selva altas. Col. Zaldívar-Riverón, Clebsch, Martínez-Salinas, Selva alta” (IB-UNAM, ZISP).

#### Description.

**Female.** Body length 3.8–5.2 mm; fore wing length 2.4–3.2 mm.

*Head*. Head width (dorsal view) 1.5–1.6 times its median length, 1.3–1.4 times width of mesoscutum. Vertex convex. Head behind eyes (dorsal view) distinctly and evenly roundness decreased; transversal diameter of eye (dorsal view) 1.4–1.7 times longer than temple. Ocelli with ocellar triangle base 1.15–1.25 times its sides; POL 1.0–1.3 times Od, 0.35–0.4 times OOL. Eye glabrous, its maximum diameter 1.3–1.4 times minimum diameter. Malar space 0.4–0.45 times maximum diameter of eye, 0.75–0.85 times basal width of mandible. Face slightly convex, its width equal to maximum diameter of eye, 1.1 times height of face and clypeus combined. Clypeal suture distinct and complete. Ventral margin of clypeus with distinct flange. Hypoclypeal depression medium-sized and rounded, its width 0.9 times the shortest distance from edge to eye, 0.4–0.45 times width of face. Occipital carina dorsally complete, not broken toward ocellar triangle, not reaching hypostomal carina and obliterated on short distance before area posterior to the mandible base. Hypostomal flange rather wide.

**Figures 1–10.**
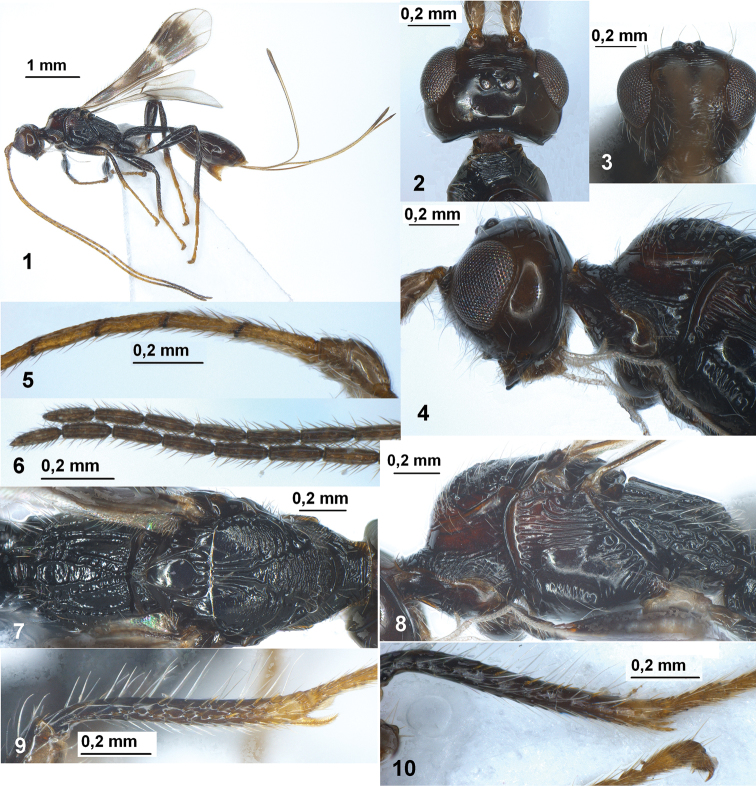
*Spathius mexicanus* sp. n. (female). **1** Habitus, lateral view **2** Head, dorsal view **3** Head, front view **4** Head and anterior half of mesosoma, lateral view **5** Basal segments of antenna **6** Apical segments of antenna **7** Mesosoma, dorsal view **8** Mesosoma, lateral view **9** Fore tibia **10** Middle tibia.

Antennae slender, almost filiform, 28–36-segmented, 1.2–1.3 times longer than body. Scape 1.6–1.7 times longer than maximum width. First flagellar segment 4.7–5.2 times longer than apical width, 1.1–1.2 times longer than second segment. Penultimate segment 3.2–3.5 times longer than width, 0.55–0.6 times as long as first segment, 0.9–1.0 times as long as apical segment; the latter subpointed or truncate apically and without spine.

*Mesosoma*. Mesosoma not depressed, maximum length 1.9–2.0 times its maximum height. Pronotal keel indistinct or fine, without posterior branch. Pronotum (dorsal view) subanteriorly with distinct transverse carina. Pronotal lateral depression shallow, delineated by carinae above and below, wide, almost entirely smooth. Mesoscutum (lateral view) slightly curvedly and highly elevated above pronotum, its median lobe (dorsal view) distinctly convex anteriorly and without anterolateral corners; mesoscutum in dorsal view 1.0–1.1 times as long as wide. Notauli complete, wide, deep anteriorly, becoming shallow posteriorly, coarsely crenulate and sometimes with fine granulation. Scutellar sulcus (prescutellar depression) deep, rather short, with three coarse carinae, finely rugulose, 0.25–0.3 times as long as scutellum. Scutellum slightly convex, with distinct lateral carinae. Subalar depression narrow, very shallow, widely and coarsely striate. Precoxal sulcus (sternaulus) about 0.5 times length of lower part of mesopleuron, slightly curved, wide, deep, distinctly and densely crenulate. Postpectal carina absent. Metanotum with short, wide and rounded apically dorsal tubercle. Metapleural flange (lobe) narrow, long, subpointed or weakly rounded apically. Propodeum without lateral tubercles.

*Wings*. Fore wing 4.0–4.1 times longer than wide. Pterostigma 4.5–5.0 times longer than its maximum width. Radial vein (r) arising behind middle of pterostigma, from its basal 0.6. Radial (marginal) cell not shortened, metacarp (R1) 1.2–1.3 times longer than pterostigma. Second radial abscissa (3RSa) 3.1–3.4 times longer than first abscissa (R) and forming with it very obtuse angle, almost as long as the weakly curved third abscissa (3RSb), almost as long as first radiomedial vein (2RS). Second radiomedial (submarginal) cell not or slightly narrowed distally, its length 3.6–3.9 times maximum width, 1.6–1.8 times length of brachial (first subdiscal) cell. Second abscissa of medial vein ((RS+M)b) rather long, 0.4–0.5 times as long as recurrent vein (m-cu). Nervulus (cu-a) postfurcal, distance between basal vein and nervulus 0.5–0.6 times nervulus length. Parallel vein (2CUb) not interstitial, arising from anterior quarter or third of distal margin of brachial (first subdiscal) cell. Mediocubital vein (M+CU) in distal half weakly curved to longitudinal anal vein (1-1A). Hind wing 5.1–5.5 times longer than its maximum width. First costal abscissa (C+Sc+R) 0.6–0.7 times as long as second abscissa (Sc+R). First abscissa of mediocubital vein (M+CU) 0.6–0.8 times as long as second abscissa (1M). Recurrent vein (m-cu) more or less distinctly sclerotised, pigmented, distinctly antefurcal, strongly oblique towards base of wing.

**Figures 11–16.**
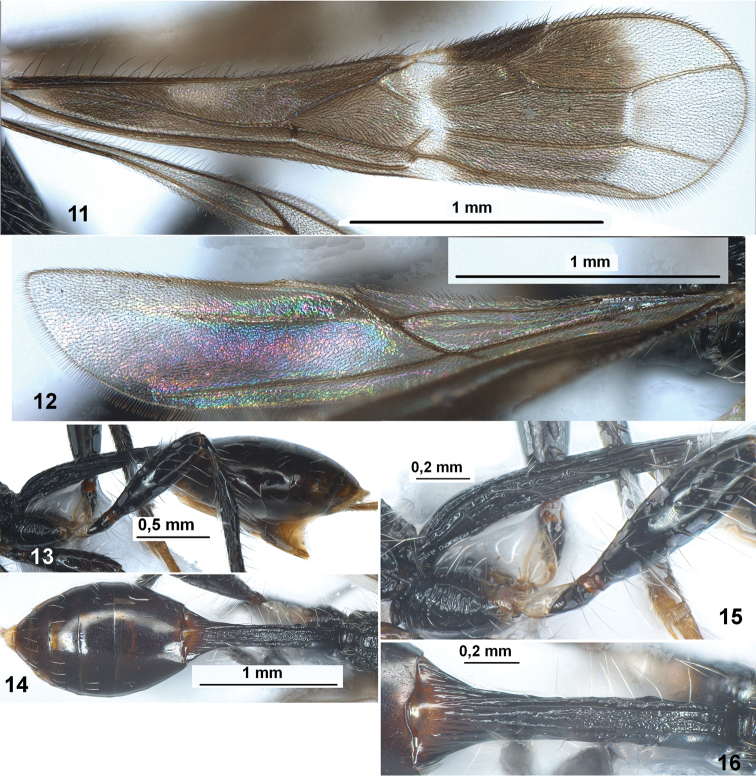
*Spathius mexicanus* sp. n. (female). **11** Fore wing **12** Hind wing **13** Metasoma, lateral view **14** Metasoma, dorsal view **15** Petiole, lateral view **16** Petiole, dorsal view.

*Legs*. Fore tibia anterior margin with long, dense spines arranged in narrow stripe. Segments of middle tarsus considerably longer than their width. Hind coxa with distinct basoventral tubercle, 1.8–2.0 times longer than maximum width. Hind femur elongate-oval, 3.2–3.5 times longer than wide. Hind tibia with outer apical lobe and two-three slender and long spines. Hind tarsus 0.9–1.0 times as long as hind tibia. Hind basitarsus 0.55–0.6 times as long as remaining segments combined. Second segment of hind tarsus 0.5–0.55 times as long as basitarsus, almost as long as thickened fifth segment (without pretarsus). Tarsal segments slightly thickened. Claws short, thick basally, with short curved apical part.

*Metasoma*. Petiole (lateral view) ventrally almost straight, dorsal basal half slightly arched and apical half nearly straight, highest in basal third or fourth; petiole slender in dorsal view, slightly widening at spiracular tubercles and distinctly apically. Length of petiole 2.9–3.1 times its apical width, 2.2–2.4 times length of propodeum; apical width about 2.0 times width at spiracle level, 2.6–2.7 times minimum subbasal width. Second tergite without laterotergites separated. Suture between second and third tergites (second suture) absent. Median length of second and third tergites combined 1.0–1.1 times its basal width, 0.6–0.65 times their maximum width. Ovipositor straight. Ovipositor sheath 2.5–2.9 times longer than petiole, 1.1–1.5 times longer than metasoma, 2.0–2.3 times longer than mesosoma, as long as or slightly longer than fore wing.

*Sculpture and pubescence*. Vertex entirely smooth. Frons densely and slightly curvedly transverse striate, with fine rugulosity between striae, sometimes almost smooth anteriorly or medioposteriorly. Face distinctly striate-rugose, smooth on median vertical area and laterally. Temple entirely smooth. Mesoscutum distinctly and densely granulate, granulation usually situated in fine semicircular subtransverse aciculation, coriaceous to almost smooth posteriorly, its medioposterior third with several distinct longitudinal striae, median lobe with dense and fine or very fine additional transverse striation; lateral lobes near notauli with distinct or coarse additional rugosity. Scutellum entirely or almost entirely smooth. Mesopleuron medially smooth, longitudinally striate in upper 0.3–0.5. Metapleuron entirely coarsely rugose-reticulate. Propodeum in anterior (dorsal or basolateral) areas almost smooth or sometimes finely reticulate, with more or less coarse additional carina along areola margins; basal (median) carina short or almost absent; areola narrow and long, 3.0–4.0 times longer than wide; petiolate area long and rather narrow, separated from areola by distinct carina; propodeum mainly coarsely transverse striate. Hind coxa entirely or almost entirely coarsely transverse striate, finely sculptured below. Hind femur entirely smooth. Petiole dorsally distinctly and more or less sparsely longitudinally striate and with distinct dense rugulosity between striae, medially rather widely and small reticulate-rugulose. Second and following tergites entirely smooth. Vertex almost entirely with very sparse, long and erect pale setae, sometimes glabrous posteriorly. Mesoscutum glabrous, with very long, sparse and almost erect yellowish setae arranged widely along margins of notauli and in single line laterally. Setae of hind tibia semi-erect, mainly long and rather dense, but dorsally in apical quarter additionally with dense and short setae; length of long setae on its dorsal surface 1.0–1.4 times maximum width of tibia.

*Colour*. Body black or dark reddish brown to reddish brown partly, head and anterior third of mesosoma dark reddish brown, occasionally head almost entirely or behind eyes yellowish brown and mesosoma red in anterior half. Antennae brownish yellow or yellow in basal third, yellow medially, dark brown to sometimes black on apical six-seven segments. Palpi pale yellow. Legs almost black or dark reddish brown, fore femur apically, always all tibiae apically and all tarsi entirely (including fifth segment) light reddish brown to brownish yellow, middle coxa and sometimes middle trochanter and hind trochanter whitish. Ovipositor sheath brownish yellow or yellow, black apically. Fore wing strongly darkened, with faintly darkened spots basally and in middle of medial cell, with very faintly darkened to almost hyaline transverse stripes in beginning of pterostigma (narrow) and on apex of wing (wide). Pterostigma dark brown to black, pale yellow to whitish yellow in basal quarter.

**Male.** Body length 2.4–4.3 mm; fore wing length 1.9–3.0 mm. Head width (dorsal view) 1.4–1.6 times its median length, 1.3–1.5 times width of mesoscutum. Head behind eyes (dorsal view) slightly roundness decreased. Malar space 0.35–0.4 times maximum diameter of eye. Face partly and finely striate-rugose, widely smooth laterally and below; its width 0.9 times maximum diameter of eye, almost equal to height of face and clypeus combined. Hypoclypeal depression width 0.5 times width of face. Antennae 26–35-segmented; brown to dark brown on apical 9–10 segments. Penultimate segment 3.7–4.3 times longer than their width, 0.6–0.7 times as long as first segment. Maximum length of mesosoma 1.9–2.1 times its maximum height. Scutellar sulcus (prescutellar depression) 0.2–0.4 times as long as scutellum. Propodeal areola sometimes with more long basal carina. Metacarp (R1) 1.25–1.35 times longer than pterostigma. Second radial abscissa (3RSa) 3.7–5.0 times longer than first abscissa (R), 1.15–1.30 times longer than first radiomedial vein (2RS). Hind wing 5.2–5.4 times longer than its maximum width. Hind femur 2.9–3.1 times longer than wide. Length of setae on dorsal surface of hind tibia 1.3–2.0 times maximum width of tibia. Hind basitarsus with distinct inner apical process. Second segment of hind tarsus 0.45–0.50 times as long as basitarsus, 0.8–0.9 times as long as fifth segment (without pretarsus). Length of petiole 3.4–3.9 times its apical width; apical width 1.8–2.0 times width at spiracle level, 2.0–2.3 times minimum subbasal width. Second-fourth tergites with laterotergites separated. Median length of second and third tergites combined 1.3–1.6 times its basal width, 0.75 times their maximum width. Sometimes body paler. Otherwise similar to female.

#### Distribution.

Mexico (Los Tuxtlas, Veracruz).

#### Diagnosis.

*Spathius mexicanus* sp. n. belongs to the *Spathius fasciatus* Walker species group. This new species is similar to the Costa Rican *Spathius albocoxus* Marsh, but differs from it by having the palpi white or pale yellow (black in *Spathius albocoxus*), mesoscutum densely granulate with striation (mainly smooth in *Spathius albocoxus*), propodeum with areola distinctly delineated by coarse carinae (often without areola in *Spathius albocoxus*), basal carina of propodeum very short or almost absent (rather long in *Spathius albocoxus*), hind coxa striate laterally (smooth in *Spathius albocoxus*), second radial abscissa about as long as first radiomedial vein (distinctly shorter in *Spathius albocoxus*), and ovipositor distinctly shorter than body (equal to body in *Spathius albocoxus*).

*Spathius mexicanus* is also similar to the Oriental *Spathius dedalus* Nixon and the Nearctic *Spathius longipetiolatus* Ashmead. However, *Spathius mexicanus* differs from *Spathius dedalus* by having the fore coxa and hind tibia mainly dark reddish brown (honey yellow or yellow in *Spathius dedalus*), fore wing strongly darkened at wide areas (faintly tinted in *Spathius dedalus*), mesoscutum distinctly granulate (finely granulate in *Spathius dedalus*), scutellum distinctly convex (almost flat in *Spathius dedalus*), propodeum with short basal carina (with long basal carina in *Spathius dedalus*), hind coxa with distinct basoventral tubercle (without basoventral tubercle in *Spathius dedalus*), second radial abscissa about as long as third abscissa (distinctly shorter than third abscissa in *Spathius dedalus*), and ovipositor distinctly shorter than body (weakly longer than body in *Spathius dedalus*). *Spathius mexicanus* differs from *Spathius longipetiolatus* by having the vertex completely smooth (strongly transversely striate in *Spathius longipetiolatus*), lateral depression of the pronotum side entirely smooth (with several oblique carinae in *Spathius longipetiolatus*), sternaulus short (long in *Spathius longipetiolatus*), propodeum with additional lateral carinae along basal carinae and fork of areola (without carinae in *Spathius longipetiolatus*), parallel vein distinctly postfurcal (nearly interstitial in *Spathius longipetiolatus*), second tergite entirely smooth (faintly shagreened at base in *Spathius longipetiolatus*), legs mainly dark (unicolourous honey yellow in *Spathius longipetiolatus*), and fore wing with wide dark brown stripe (subhyaline in *Spathius longipetiolatus*).

### Spathius chamelae

Taxon classificationAnimaliaHymenopteraBraconidae

Belokobylskij & Zaldívar-Riverón sp. n.

http://zoobank.org/ABE57550-B9E7-446F-BF87-10AC21D95A28

Figs 17 –30

#### Type material.

Holotype: female, “México, Jalisco, Est. Chamela. Camino a Calandria, 19.50485N, 105.03786W, 45 m. 18–20.XI.2009. Platos amarillos, Selva baj. cad. Cham-034, Alejandro Zaldívar R.", voucher no. BOLD ASDOR 433, GenBank accession no. (COI) HM434538 (IB UNAM).

Paratypes. 1 male, same data as holotype, voucher no. BOLD ASDOR 432, GenBank accession no. (COI) HM434537 (ZISP); 1 male, “México, Jalisco, Est. Chamela. Camino a Calandria, 19.504N, 105.037W, 52 m. 04.XI.2009. Red de barrido. Selva baja mediana. Cham-019, H. Clebsch / A. Zaldívar", voucher no. BOLD ASDOR 371, GenBank accession no. (COI) HM434515 (IB UNAM); 1 female, “México, Jalisco, Est. Chamela. Camino a Calandria, 19.504N, 105.037W, 45 m. 3–5.IX.2009. Platos amarillos, Selva baj. cad. Cham-013, H. Clebsch / A. Zaldívar R.", voucher no. BOLD ASDOR: 373, GenBank accession no. (COI) HM434517 (ZISP); 1 female, same data, voucher no. BOLD ASDOR: 372, GenBank accession no. (COI) HM434516 (IB UNAM); 1 male, same data, voucher no. CHAM-13-Spath-X-4, ASDOR 375, GenBank accession no. (COI) HM434 (IB UNAM); 1 male, “México, Jalisco, Chamela Fund. Cuixmala El sendero, 19.419N, 104.973W, 61 msnm. 7–IX–2009. Red de barrido. Selva baj. cad. Cham-023, H. Clebsch / A. Zaldívar R." (ZISP).

#### Description.

**Female.** Body length 3.4–4.3 mm; fore wing length 2.2–2.7 mm.

*Head*. Head width (dorsal view) 1.5 times its median length, 1.3–1.4 times width of mesoscutum. Vertex convex. Head behind eyes (dorsal view) distinctly and evenly roundness decreased; eye transverse diameter (dorsal view) 1.25–1.4 times longer than temple. Ocelli with ocellar triangle base 1.2–1.3 times its sides; POL 1.6–2.2 times Od, 0.45–0.5 times OOL. Eye with sparse and rather distinct setae, maximum diameter of eye 1.2 times its minimum diameter. Malar space 0.45–0.5 times maximum diameter of eye, 0.8–1.0 times basal width of mandible. Face slightly convex, its width 1.1–1.2 times maximum diameter of eye, 1.1–1.2 times height of face and clypeus combined. Clypeal suture distinct and complete. Ventral margin of clypeus with distinct flange. Hypoclypeal depression medium-sized and rounded, its width 0.8–1.0 times the shortest distance from edge to eye, 0.4–0.5 times width of face. Occipital carina dorsally complete, not broken toward ocellar triangle, not reaching hypostomal carina and obliterated on short distance before area posterior to the mandible base, sometimes fused with hypostomal carina by additional ruga. Hypostomal flange rather wide.

**Figures 17–26.**
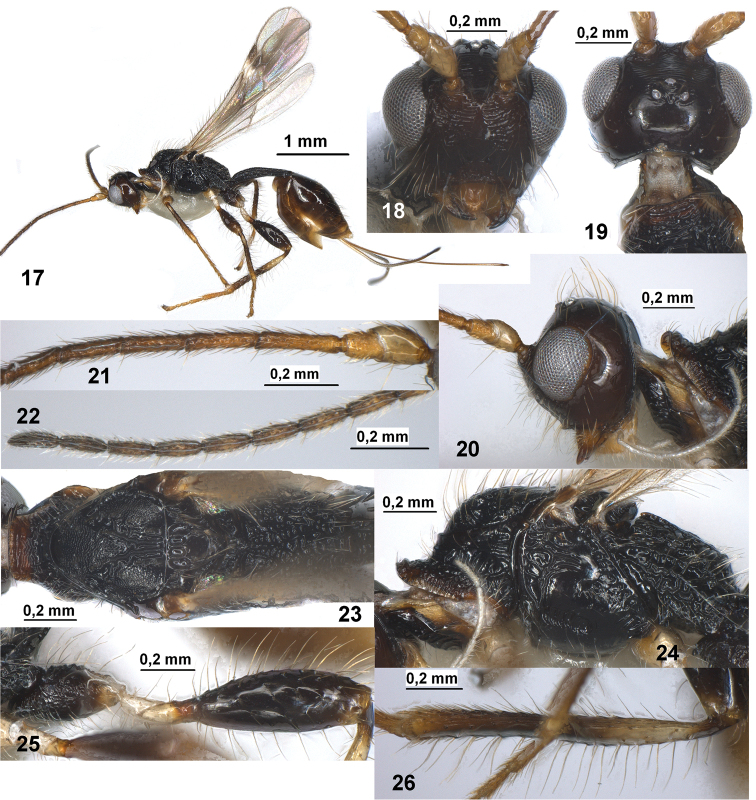
*Spathius chamelae* sp. n. (female). **17** Habitus, lateral view **18** Head, front view **19** Head, dorsal view **20** Head and anterior part of mesosoma, lateral view **21** Basal segments of antenna **22** Apical segments of antenna **23** Mesosoma, dorsal view **24** Mesosoma, lateral view **25** Hind coxa and femur **26** Hind tibia.

Antennae slender, almost filiform, 30–31-segmented, 1.1–1.2 times longer than body. Scape 1.5–1.6 times longer than maximum width. First flagellar segment 4.0–4.6 times longer than its apical width, 1.1–1.2 times longer than second segment. Penultimate segment 2.5–2.8 times longer than their width, 0.55 times as long as first segment, 0.8–0.9 times as long as apical segment, the latter subpointed apically and without spine.

*Mesosoma*. Mesosoma not depressed, maximum length 2.0–2.1 times its maximum height. Pronotal keel distinct, its posterior branch distinct, not fused and at least slightly separated from posterior margin of pronotum. Pronotum (dorsal view) subanteriorly with high and thick transverse carina. Pronotal lateral depression shallow, delineated by carinae below or upper and below, wide, entirely coarsely and densely transverse crenulate. Mesoscutum (lateral view) slightly curvedly and highly elevated above pronotum, its median lobe (dorsal view) distinctly convex anteriorly and without anterolateral corners; mesoscutum in dorsal view about as long as wide. Notauli complete, wide, deep anteriorly, slightly shallow posteriorly, coarsely irregularly crenulate. Scutellar sulcus (prescutellar depression) deep, long, with three coarse carinae, almost smooth between carinae, 0.45–0.5 times as long as scutellum. Scutellum slightly convex, with fine lateral carinae. Subalar depression narrow, shallow, widely and coarsely rugose-striate. Precoxal sulcus (sternaulus) about half length of lower part of mesopleuron, slightly curved, wide, deep, distinctly and sparsely crenulate. Postpectal carina absent. Metanotum with short, wide and pointed apically dorsal tubercle. Metapleural flange (lobe) narrow, long, slightly rounded apically. Propodeum without lateral tubercles.

*Wings*. Fore wing 4.0–4.3 times longer than wide. Pterostigma 3.8-4.0 times longer than its maximum width. Radial vein (r) arising behind middle of pterostigma, from its basal 0.65–0.7. Radial (marginal) cell not shortened, metacarp (R1) 1.5–1.6 times longer than pterostigma. Second radial abscissa (3RSa) 3.0–5.0 times longer than first abscissa (R) and forming with it obtuse angle, 0.55–0.6 times as long as the almost straight third abscissa (3RSb), 1.1–1.4 times longer than first radiomedial vein (2RS). Second radiomedial (submarginal) cell not narrowed distally, its length 3.0–3.7 times maximum width, 1.3–1.5 times length of brachial (first subdiscal) cell. Second abscissa of medial vein ((RS+M)b) rather or very short, 0.1–0.3 times as long as recurrent vein (m-cu). Nervulus (cu-a) postfurcal, distance between basal vein and nervulus 0.2–0.3 times nervulus length. Parallel vein (2CUb) not interstitial, arising from anterior third of distal margin of brachial (first subdiscal) cell. Mediocubital vein (M+CU) in distal half weakly curved to longitudinal anal vein (1-1A). Hind wing 5.6–6.0 times longer than its maximum width. First costal abscissa (C+Sc+R) about 0.5 times as long as second abscissa (Sc+R). First abscissa of mediocubital vein (M+CU) 0.5–0.6 times as long as second abscissa (1M). Recurrent vein (m-cu) more or less unsclerotised, faintly pigmented, slightly antefurcal, strongly oblique towards base of wing.

**Figures 27–30.**
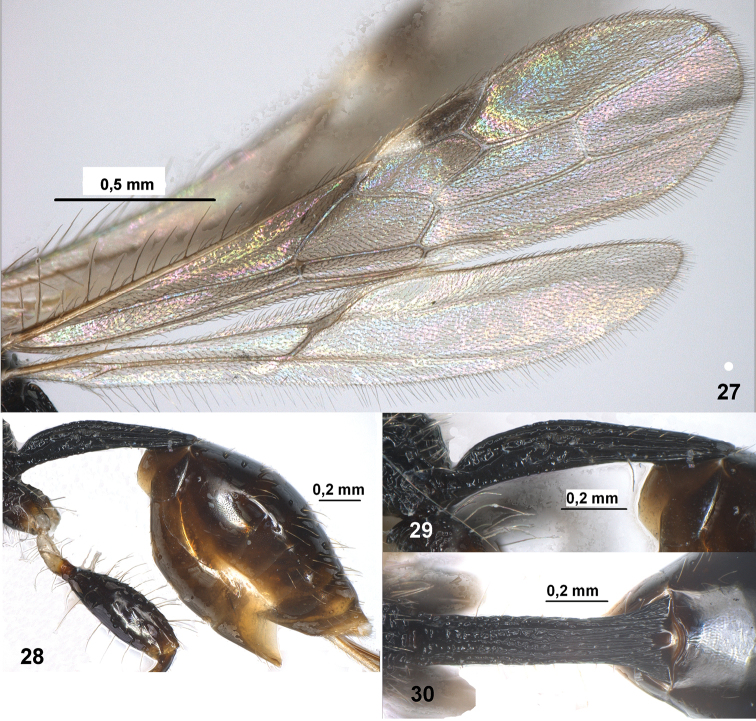
*Spathius chamelae* sp. n. (female). **27** Fore and hind wings **28** Metasoma, lateral view **29** Petiole, lateral view **30** Petiole, dorsal view.

*Legs*. Fore tibia anterior margin with rather long, dense spines arranged in narrow stripe. Segments of middle tarsus longer than their width. Hind coxa with distinct basoventral tubercle, 1.8–2.0 times longer than maximum width. Hind femur elongate-oval, 2.9–3.1 times longer than wide. Hind tibia with outer apical lobe having two-three slender and short spines. Hind tarsus 0.9 times as long as hind tibia. Hind basitarsus with distinct ventral carina, 0.55–0.6 times as long as remaining segments combined. Second segment of hind tarsus 0.55 times as long as basitarsus, 1.1–1.2 times longer than fifth segment (without pretarsus). Tarsal segments slightly thickened. Claws short, thick basally, with short curved apical part.

*Metasoma*. Petiole (lateral view) ventrally slightly curved, dorsal distinctly and almost evenly arched, in apical half nearly straight, highest near middle; in dorsal view petiole slender, slightly widened at spiracular tubercles and distinctly widened apically. Length of petiole 2.8–2.9 times its apical width, about 2.0 times length of propodeum; apical width about 1.8 times minimum subbasal width. Second tergite without laterotergites separated. Suture between second and third tergites (second suture) absent. Median length of second and third tergites combined 1.2–1.4 times its basal width, 0.7–0.8 times their maximum width. Ovipositor almost straight. Ovipositor sheath 2.5–2.9 times longer than petiole, 1.2–1.3 times longer than metasoma, 1.8–2. times longer than mesosoma, almost as long as fore wing.

*Sculpture and pubescence*. Vertex entirely smooth. Frons densely and slightly curvedly transverse striate, without rugulosity between striae, smooth laterally. Face distinctly striate, with fine rugulosity between striae, smooth latero-ventrally. Temple entirely smooth. Mesoscutum distinctly and densely granulate-reticulate, finely reticulate-coriaceous or sometimes almost smooth posteriorly, its medioposterior third with several distinct longitudinal striae, median lobe laterally and anteriorly with dense and fine transverse striation; lateral lobes near notauli with short and sparse additional rugosity. Scutellum mainly smooth, sometimes finely reticulate-coriaceous or rugulose laterally. Mesopleuron medially smooth, striate in upper 0.2–0.3 and anteriorly, rugulose posteriorly. Metapleuron entirely coarsely rugose-reticulate. Propodeum in anterior (dorsal or basolateral) areas entirely rugose or rugulose, usually with coarse additional carina along areola margins; basal (median) carina rather long; areola narrow and long, 2.0–2.3 times longer than wide; petiolate area long and rather narrow, separated from areola by distinct carina; propodeum mainly coarsely rugose-striate. Hind coxa rugose in dorsal half and with additional transverse striation dorso-posteriorly, finely rugulose to smooth in ventral half. Hind femur smooth, sometimes partly finely striate dorsally. Petiole dorsally distinctly and densely longitudinally striate with distinct dense rugulosity between striae in basal half, medially usually reticulate-rugulose. Second and following tergites entirely smooth. Vertex almost entirely with very sparse, long and erect pale yellowish setae, glabrous medially. Mesoscutum with very long, sparse and almost erect yellowish setae arranged widely along margins of notauli and in single line laterally. Setae of hind tibia almost erect, mainly long and rather sparse, but dorsally in apical quarter additionally with very sparse and short setae; length of long setae 1.5–2.0 times maximum width of tibia.

*Colour*. Body black, head mainly and mesosoma anteriorly dark reddish brown or partly reddish brown, metasoma behind petiole dark reddish brown, paler laterally, brownish yellow apically; occasionally (in small specimens) body almost entirely reddish brown. Antennae dark reddish brown or reddish brown, almost black apically, without pale subapical segments. Palpi pale yellow or whitish yellow. Legs mainly almost black or reddish brown, fore and middle coxae yellow, all trochanters and trochantelli white or pale yellow, most part of fore femur, base and apex of middle femur (sometimes), apical 0.3–0.4 of fore and middle tibiae and their subbasal short areas, and all tarsi yellow or brownish yellow; hind tibia subbasally in wide area whitish yellow, apically yellow. Ovipositor sheath pale yellow to yellow, black apically. Fore wing infuscate, with narrow hyaline transverse stripe crossing beginning of pterostigma. Pterostigma almost black, pale yellow to whitish yellow in basal third and apically.

**Male.** Body length 2.3–3.3 mm; fore wing length 1.6–2.2 mm. Antennae 29–32-segmented. Penultimate segment 3.3–3.8 times longer than their width. Mesoscutum distinctly and densely granulate almost entirely. Propodeal areola short, 1.8–2.0 times longer than maximum width. Second radial abscissa (3RSa) 3.0–3.8 times longer than first abscissa (R), 1.1–1.2 times longer than first radiomedial vein (2RS). Hind femur 2.6–2.8 times longer than wide. Hind basitarsus with distinct inner apical process. Second segment of hind tarsus as long as fifth segment (without pretarsus). Length of petiole 3.0–4.0 times its apical width, 2.3–2.5 times longer than propodeum. Second and sometimes basal half of third tergites with laterotergites separated. Median length of second and third tergites combined 1.8–2.0 times its basal width, 0.8–0.9 times their maximum width. Otherwise similar to female.

#### Distribution.

Mexico (Chamela, Jalisco).

#### Comparative diagnosis.

*Spathius chamelae* sp. n. belongs to the *Spathius fasciatus* Walker species group. This new species is very similar to *Spathius mexicanus* sp. n., but differs from it in having the POL distinctly larger than Od (shorter in *Spathius mexicanus*); eye with sparse and short setae (glabrous in *Spathius mexicanus*); pronotal keel distinct, with its posterior branch present and not fused with posterior margin of pronotum (indistinct or fine and without its posterior branch in *Spathius mexicanus*); pronotal lateral depression entirely coarsely transverse crenulate (almost entirely smooth in *Spathius mexicanus*); pterostigma wider (narrower in *Spathius mexicanus*); second radial abscissa (3RSa) distinctly longer than first radiomedial vein (2RS) (almost equal in *Spathius mexicanus*); petiole (lateral view) dorsal distinctly arched (slightly arched in *Spathius mexicanus*); basolateral areas of propodeum entirely rugose, basal (median) carina rather long, and areola less narrow (smooth, basal carina almost absent and areola narrow in *Spathius mexicanus*); setae on hind tibia long (shorter in *Spathius mexicanus*); hind tibia subbasally in wide area whitish yellow (mainly almost black in *Spathius mexicanus*); fore wing faintly infuscate (strongly infuscate in *Spathius mexicanus*).

## Supplementary Material

XML Treatment for Spathius mexicanus

XML Treatment for Spathius chamelae

## References

[B1] Belokobylskij SA (1992) On the classification and phylogeny of the braconide wasps of subfamilies Doryctinae and Exothecinae (Hymenoptera, Braconidae). Part I. On the classification, 1. Entomologicheskoe obozrenie 71: 900–928 [in Russian]

[B2] Belokobylskij SA (2003) The species of the genus *Spathius* Nees, 1818 (Hymenoptera: Braconidae: Doryctinae) not included in the monograph by Nixon (1943). Annales Zoologici 53: 347–488

[B3] Belokobylskij SA Maeto K (2009) Doryctinae (Hymenoptera, Braconidae) of Japan. Fauna Mundi. Volume 1 Warshawska Drukarnia Naukowa, Warszawa, 806 pp

[B4] Cauich-Kumul R Delfin-Gonzalez H Lopez-Martinez V Sharkey M (2012) Braconid wasps (Hymenoptera: Braconidae) of northern Yucatan, Mexico: subfamilies Agathidinae and Doryctinae (excluding *Heterospilus* Haliday). Journal of Kansas Entomological Society 85: 186–205. doi: 10.2317/JKES120212.1

[B5] Ceccarelli SF Sharkey MJ Zaldívar-Riverón A (2012) Species identification in the taxonomically neglected, highly diverse, Neotropical parasitoid wasp genus *Notiospathius* (Braconidae: Doryctinae) based on an integrative molecular and morphological approach. Molecular Phylogenetics and Evolution 62: 485–495. doi: 10.1016/j.ympev.2011.10.018 22079550 10.1016/j.ympev.2011.10.018

[B6] Chen J Shi Q (2004) Systematic studies on Doryctinae of China (Hymenoptera: Braconidae). Fujian Science and Technology Publishing House, Fujian, 274 pp

[B7] Coronado-Blanco JM (2013) La familia Braconidae (Hymenoptera) en Mexico. Entomologia Mexicana 12: 31–46

[B8] Marsh PM (2002) The Doryctinae of Costa Rica (excluding the genus *Heterospilus* Haliday). Memoirs of the American Entomological Institute 70: 1–319

[B9] Marsh PM Strazanac JS (2009) A taxonomic review of the genus *Spathius* Nees (Hymenoptera: Braconidae) in North America and comments on the biological control of the emerald ash borer (Coleoptera: Buprestidae). Journal of Hymenoptera Research 18(1): 80–112

[B10] Nixon GEJ (1943) A revision of the Spathiinae of the Old World (Hymenoptera, Braconidae). Transactions of the Royal Entomological Society of London 93: 173–495. doi: 10.1111/j.1365-2311.1943.tb00434.x

[B11] Sharkey MJ Wharton RA (1997) Morphology and terminology. In: Wharton RA Marsh PM Sharkey MJ (Eds) Manual of the New World genera of the family Braconidae (Hymenoptera). Special Publication No 1 International Society of Hymenopterists, Washington, 21–40

[B12] Shenefelt RD Marsh PM (1976) Hymenopterorum Catalogus. Pars 13. Braconidae 9. Doryctinae. Dr W. Junk, ’s-Gravenhage, 1263–1424

[B13] Yu DS van Achterberg C Horstman K (2012) Taxapad 2012, Ichneumonoidea 2011. Database on flash-drive. Ottawa, Ontario, Canada

[B14] Zaldívar-Riverón A Belokobylskij SA León-Regagnon V Briceño R Quicke DLJ (2008) Molecular phylogeny and historical biogeography of the cosmopolitan parasitic wasp subfamily Doryctinae (Hymenoptera: Braconidae). Invertebrate Systematics 22: 345–363. doi: 10.1071/IS07028

[B15] Zaldívar-Riverón A Martínez JJ Ceccarelli SF De Jesús-Bonilla VS Rodríguez-Pérez AC Reséndiz-Flores A Smith MA (2010) DNA barcoding a highly diverse group of parasitoid wasps (Braconidae: Doryctinae) from a Mexican nature reserve. Mitochondrial DNA 21(S1): 18–23. doi: 10.3109/19401736.2010.523701 21271854 10.3109/19401736.2010.523701

